# Scar Epilepsy as a Complication of Subarachnoid Hemorrhage in a Patient With Adult Polycystic Kidney Disease: A Case Report

**DOI:** 10.7759/cureus.41537

**Published:** 2023-07-07

**Authors:** Adil Khan, Maryem Anwar, Farah N Zaidi, Shaima Ghabsha, Anees ur Rehman

**Affiliations:** 1 Internal Medicine, Khyber Medical College, Peshawar, PAK; 2 Family Medicine, National Health Service (NHS), Slough, GBR; 3 Medicine, Queen Elizabeth Hospital, King's Lynn, GBR; 4 Gastroenterology, Royal Bournemouth Hospital, Bournemouth , GBR; 5 Medicine, Ayub Medical College, Abbottabad, PAK

**Keywords:** generalized tonic-clonic seizures, computed tomography angiogram, berry aneurysm, aneurysmal subarachnoid hemorrhage, polycystic kidney disease (pkd)

## Abstract

Polycystic kidney disease (PKD) is the most common hereditary disorder of kidneys. In adults, PKD1 gene mutation almost always signifies its subtype, autosomal dominant polycystic kidney disease (ADPKD), or adult polycystic kidney disease. ADPKD is a multisystemic disorder giving rise to renal and extra-renal manifestations. The renal shutdown is the most feared renal complication while the development of intracranial aneurysms is considered the most lethal extra-renal feature. This can be attributed to the increased risk of rupture associated with aneurysms leading to a condition called subarachnoid hemorrhage (SAH). While being notorious for the subtle situations SAH often leads to, its association with the onset of seizures is a matter of high clinical significance. We present a patient with a kidney disorder (ADPKD) that has led to the onset of epilepsy. Five years after the diagnosis of ADPKD, he developed an aneurysm in the right internal carotid artery, for which he was treated conservatively. After four months, he presented with the onset of symptoms of SAH, which was confirmed by computed tomography angiography. Clipping was unable to be performed, and the patient was treated conservatively, this time as well. Recently, the patient presented with the onset of generalized tonic-clonic seizures, unable to be controlled with single anti-epileptics. He was stabilized by dual intravenous antiepileptics but on further workup, he was found to have a recurrence of a berry aneurysm for which he was referred to a neurosurgeon for a clipping procedure to be performed. The operation was successful, but the patient was still found to be an epileptic for which he was discharged with a long-term course of double anti-epileptics.

## Introduction

Autosomal dominant polycystic kidney disease (ADPKD) is a single-parent inherited disorder. It is mainly diagnosed in adulthood; therefore, it is also called adult polycystic kidney disease (PKD). It is characterized by the formation of fluid-filled cysts on the surface of the kidneys leading to the deterioration of its function [1]. Rather than being an exclusive disease of the kidney, ADPKD is a multisystemic disorder, with the main extra renal features being cyst formation in the liver, pancreas, and brain, intracranial aneurysms (ICAs), abdominal hernias, and cardiac complications. Among these, aneurysms are considered to be one of the fairly lethal complications [2]. An aneurysm in the brain is formed due to thinning of an arterial wall (berry aneurysm) which in most cases remains asymptomatic and is diagnosed only after its rupture leading to a severe type of headache. This condition is called subarachnoid hemorrhage (SAH) [3]. In rare cases, the rupture of the vascular wall may lead to the formation of scar tissue exerting physical pressure on the brain and damaging the neurons. This may insinuate manifestations of focal or generalized seizures. This can be regarded as scar epilepsy or post-SAH epilepsy [4].

Here, we report a case of adult PKD in which renal damage led to hypertension and the formation of a berry aneurysm in the right internal carotid artery. Ultimately, its rupture led to SAH, and the patient presented with epilepsy and recurrence of a berry aneurysm, four months after the incident.

## Case presentation

A 45-year-old male was brought to the emergency and accident department with uncontrolled fits. He was salivating and unconscious, with a Glasgow coma scale (GCS) of 4; the episode lasted for four minutes. As the patient described, he regained consciousness afterward but was feeling confused and had no clear memory of the attack. The patient had no associated vomiting, fever, or urinary incontinence.

**Figure 1:**
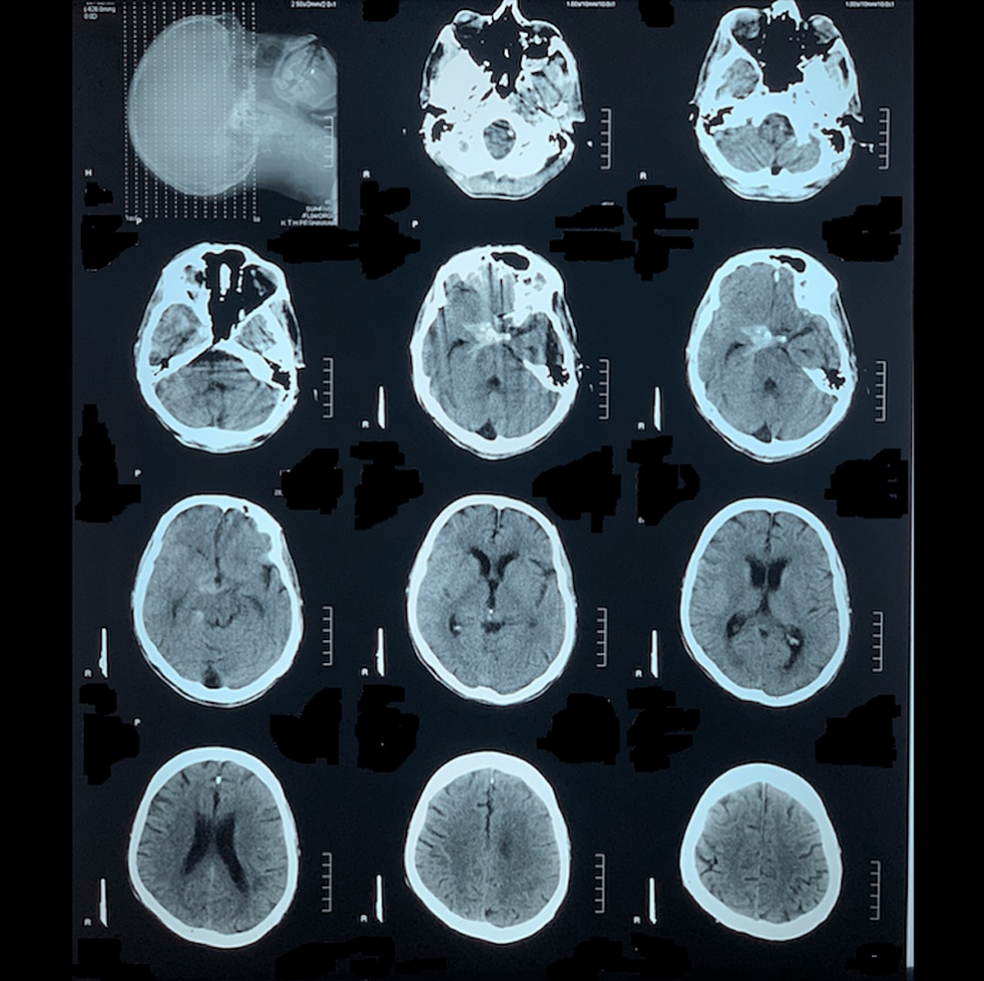
Computed tomography (CT) scan without contrast, findings of hyper-densities along the basal cisterns and right Sylvian fissure (more marked in suprasellar cistern).

There was no history of any head injury, infections, drug abuse, or familial tendency. He was diagnosed with adult PKD five years ago. At that time, he presented to the nephrology outpatient department with complaints of bilateral renal pain, gross hematuria, and abdominal bloating. He had no history of any inherited kidney diseases in the family. On further workup, his creatinine levels were found to be high due to which he was considered to be in a state of acute kidney injury. An abdominal ultrasound showed multiple cysts over the surface of both kidneys. Furthermore, genetic sequencing found the PKD1 gene to be positive. All these findings led to the confirmation of ADPKD. Two repeat dialysis were performed at the time and the patient was stabilized. Ever since then, the patient was kept on supplemental drug therapy for chronic kidney disease (CKD). The therapy included calcium acetate tablets (to bring down high phosphate levels), vitamin D, and iron supplements. Moreover, ramipril, (an angiotensin-converting enzyme inhibitor) was used for high blood pressure, and a routine of weekly hemodialysis was followed strictly. Being middle-aged and a chronic hypertensive, he was considered a suitable candidate for screening for ICAs and was put on regular computed tomography angiography (CTA) scans which he underwent regularly, on his visits to the hospital. The patient was quite functional with the treatment, until eight months ago, when a berry aneurysm, measuring approximately 4.3x3.1mm in size, was diagnosed at the right internal carotid artery, on his routine CTA. The preferred choice of treatment was to clip the aneurysm, but the patient declined the procedure. Hence, he was treated conservatively with careful observation to prevent risk factors, but no intervention was performed because the patient was already suffering from CKD. Four months ago, the patient presented with an acute history of severe headache. On computed tomography (CT) scan without contrast, findings of hyper-densities along the basal cisterns and right Sylvian fissure (more marked in the suprasellar cistern) were found. Evidence of scar formation as a compensatory mechanism was also found around the affected blood vessels. These findings were highly suggestive of SAH (Figure 1). The diagnosis was confirmed by CTA.

Here again, clipping was the treatment of choice but could not be performed as the patient was unstable and for the same reason, high-risk consent was declined by the attendants of the patient. To prevent vasospasms after SAH, the patient was treated orally with nimodipine (a calcium channel blocker) 60mg, four hourly for 20 days. Recently (after about four months after the incident of SAH), the patient developed seizures which were generalized tonic-clonic in nature, not responding to any individual anti-epileptics used. On performing the baseline investigations, the deteriorating function of the kidneys was evident, depicted by the low hemoglobin and hematocrit and increased blood urea, creatinine, sodium, and potassium (Tables 1, 2).

**Table 1:** Baseline CBC and CRP HCT: Hematocrit; MCV: mean corpuscular volume; CRP: C-reactive protein; CBC: complete blood count; WBC: white blood cell; RBC: red blood cell

Parameter	Values: Day 1	Day 2	Day 3	Reference
WBC	10.6	9.8		4.5-11x10^9/L
Hemoglobin	8.5	7.8	8.9	14-16.5 g/dl
RBC	2.93	3.4	3.6	4.45x10^12/L
HCT	24.7	26.4	27	36-54%
MCV	84.4	92	88.9	76-96fL
Platelets	122	130	143	150-400x10^9/L
CRP	4.5	3.4	4.9	Less than 5.0 mg/L

**Table 2:** Renal Function Tests

Parameter	Values: Day 1	Day 2	Day 3	Reference
Blood Urea	189.8	194.6	188	10-50 mg/dL
Osmolarity	350	379	365	280-295 mmol/L
Creatinine	8.76	7.9	7.89	0.64-1.2 mg/dL
Creatinine clearance	35	34	57	97-137 ml/min
Sodium	126.2	130.0	134.1	135-150 mmol/L
Potassium	5.5	6	5.4	3.5-5.1 mmol/L
Chloride	96.1	99.7	109	96-112 mmol/L

**Figure 2:**
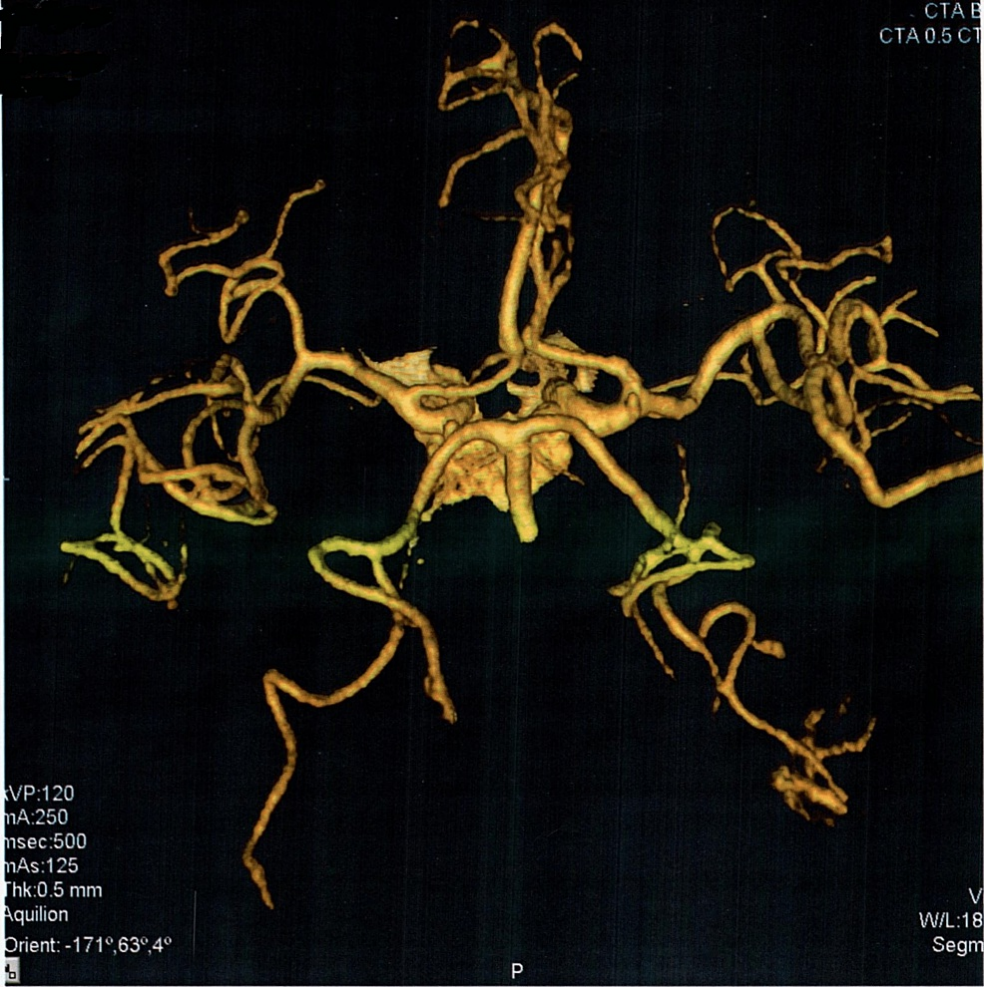
Computed tomography angiogram indicating cerebral blood flow

**Figure 3:**
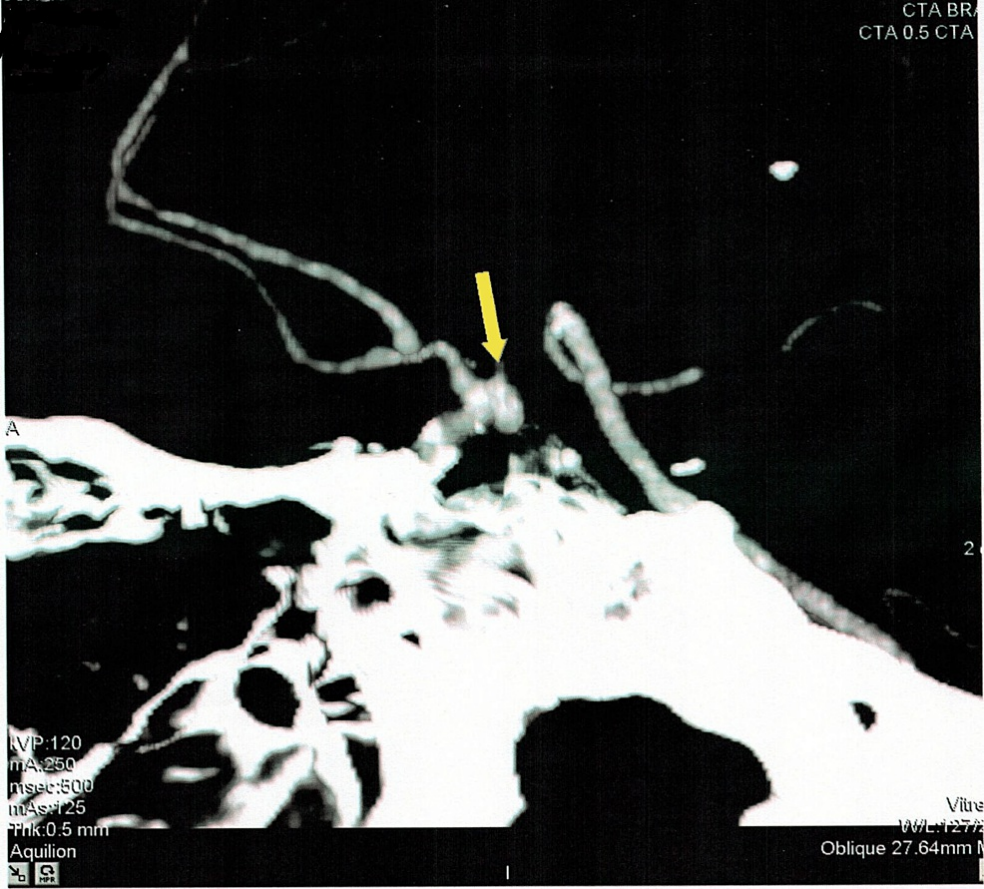
Computed tomography angiogram depicting the location of the aneurysm

**Figure 4:**
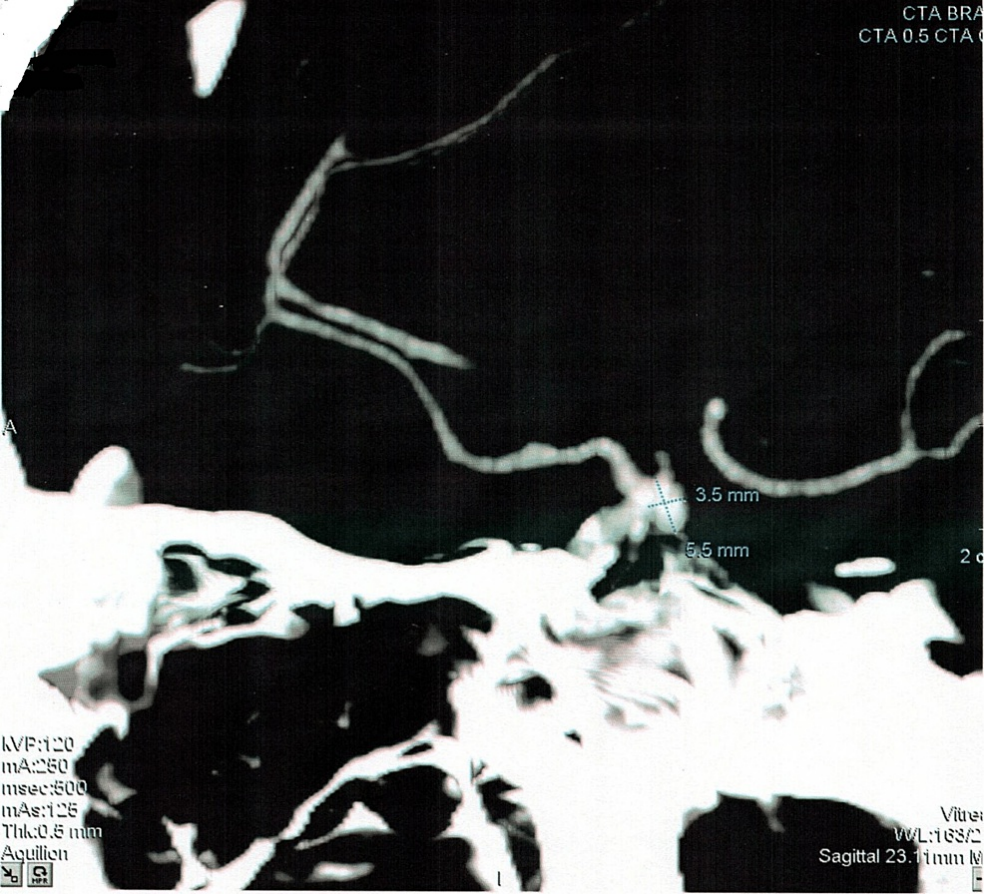
Computed tomography angiogram depicting the size of the aneurysm

**Figure 5:**
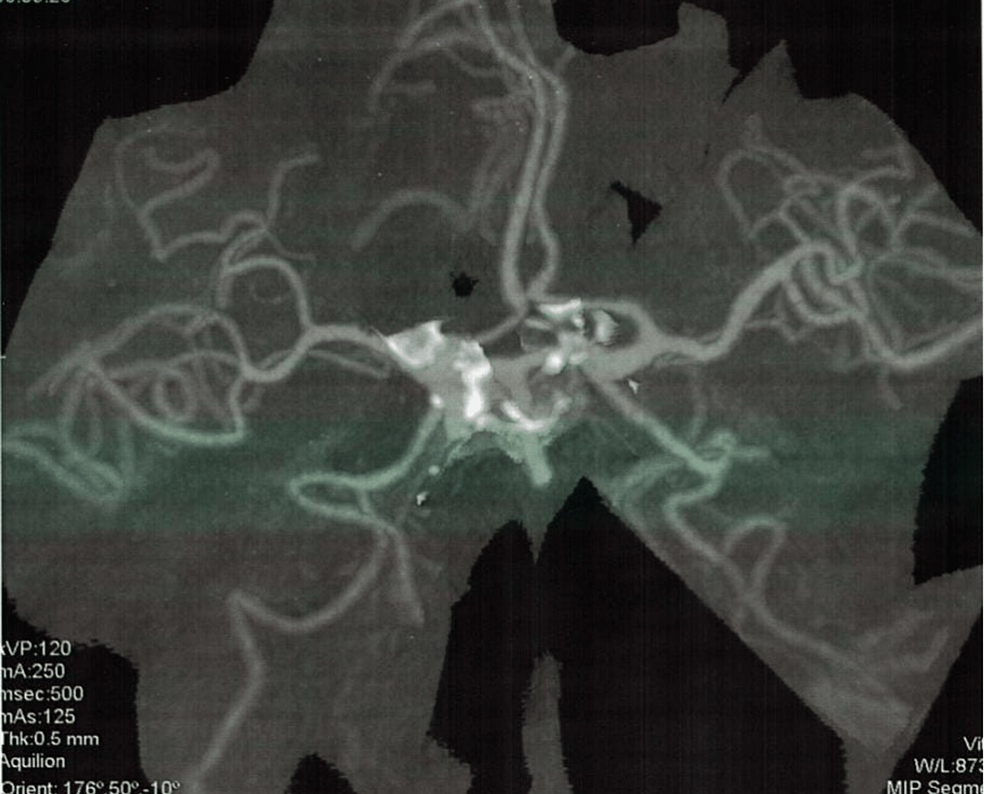
Computed tomography angiogram depicting hyper-densities

**Figure 6:**
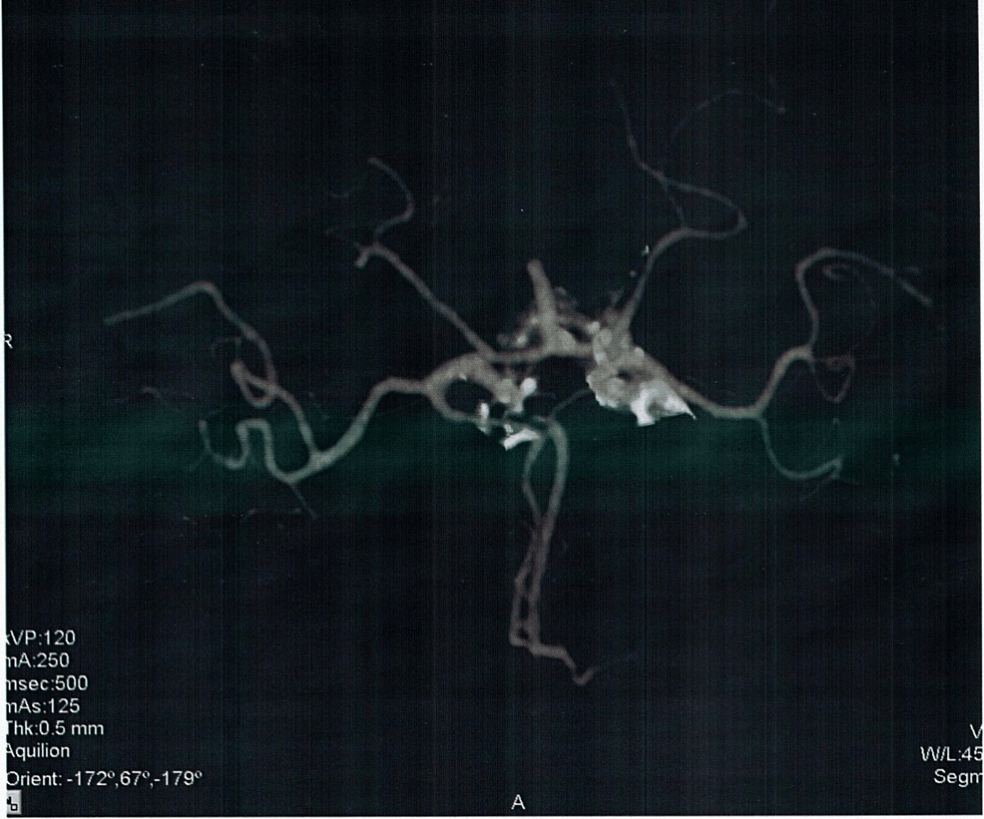
Computed tomography angiogram depicting hyper-densities

On further workup by CTA, another berry aneurysm of the size 5.5x3.5mm was evident at the same location as the previous one (right internal carotid artery). Evidence of scar formation as a compensatory mechanism was also found around the affected blood vessels. The radiological findings are provided in Figures 2-6.

Ultimately, the patient was put on double intravenous (IV) anti-epileptics (sodium valproate and levetiracetam), and the routine hemodialysis was performed. After stabilization, the patient was referred to neurosurgery for the clipping to be performed. The procedure was successful, but the patient was still found to be prone to development of seizures, for which he was discharged with an oral course of double anti-epileptics, sodium valproate, and levetiracetam. Regular follow-up was advised after every two weeks, and the patient was stable on follow-up.

## Discussion

PKD is the commonest form of hereditary kidney disease, with the autosomal dominant as the commonest subtype [1,2,5]. ADPKD is associated with a mutation in PKD1 and PKD2 genes. PKD1 is present in 75% of cases of ADPKD [5]. Along with genetic mutation, family history is considered one of the most common determining factors of diagnoses and disease progression. The common age of presentation of ADPKD is greater than 30 with a specific sex predilection toward males [6]. Our patient, being a known case of adult PKD, was also positive for PKD1 mutation. He had the disease diagnosed at the age of 40. However, the family history was negative.

ADPKD leads to various renal and extra-renal complications. The renal complications include cyst formation, gross hematuria, renal stone formation, hypertension, and ultimately, end-stage renal disease [7]. ADPKD is the commonest genetic cause of renal failure [5]. Our patient being hypertensive (since the diagnosis of PKD) and having CKD with gross hematuria and bilateral flank pain also affirms the findings above. However, he did not complain of renal stone formations. Management of CKD ultimately requires hemodialysis or renal transplant as the definitive treatment but before this stage is reached, advances have been made to manage and slow down the deteriorating renal function. It can mainly be achieved by preventing complete loss of renal function and controlling blood pressure. For this purpose, the use of tolvaptan for preventing renal shutdown and controlling blood pressure with renin-angiotensin system inhibitors or angiotensin-converting enzyme (ACE) inhibitors [8]. Our patient after initial stabilization was put on supplemental therapy for CKD, including calcium acetate (to prevent high potassium levels), vitamin D, and iron supplements. For blood pressure, an ACE inhibitor, ramipril, was prescribed. Furthermore, weekly sessions of hemodialysis were performed regularly.

The extra-renal complications of ADPKD as compiled by Luciano et al. and Pirson are composed of formation of cysts in areas other than the kidneys, vascular, cardiac abnormalities, abdominal hernias, etc. [2,9]. Hepatic cyst formation is the most prevalent extra-renal manifestation of ADPKD [9]. However, not as frequent as hepatic cysts, the most lethal of these complications is the development of ICAs. Presenting without a family history, it is a finding in almost 6% of patients with ADPKD [2]. Its ability to remain symptom-free, high incidence of recurrence, and risk of rupture (SAH) contribute to its severity [2,9]. After initial presentation with symptoms of SAH, our patient, on further workup, was found positive for remnants of berry aneurysm in the right carotid artery. Familial history and previous history of ruptured aneurysms are the greatest risk factors for SAH [10]. Our patient having a positive history of the occurrence of SAH in the past (four months ago from the latest incident of epilepsy) confirms one of the risk factors mentioned because a recent CTA found another berry aneurysm occurring again in the right internal carotid artery.

Rare among the findings mentioned above is a complication of the development of seizures, as an ultimate impediment of a hereditary renal disease (ADPKD). Of all the post-SAH patients, 25% will develop epilepsy or seizures, as mentioned by Marigold et al. It may be due to the blood itself causing nerve damage or formation of edema or scar around the injured vessel [11]. This was the main finding in our patient, who presented with generalized tonic-clonic seizures four months after the incident of SAH. After the SAH, the presence of scar tissue was also confirmed by CT at the area of remnants of berry aneurysm, located on the right internal carotid artery.

There are no specific guidelines for treating a patient with seizures developing after SAH. As SAH is an emergency with a high mortality (45-50%), the prevention of this condition can be considered as the most effective strategy [2,10]. For this purpose, regular screening is the key. A review of different studies by Rinkel et al. states that the patients with ADPKD are one of the most suitable candidates for screening of SAH and magnetic resonance angiography is considered the most preferable procedure for screening [12]. However, we used CTA as the main method of diagnosis of SAH in our patient. When an aneurysm is confirmed, a multi-discipline team including a neurologist, a nephrologist, and a medical specialist should be engaged for the preferred method of treatment. Options include surgical (clipping, coiling, or endovascular therapy) and conservative (nimodipine use) procedures. It is because even if some procedures are of vital importance in saving lives, these are not without certain risks [2,9,10]. Hence, clinical studies on the outcome of various treatment options used for patients having a high risk of aneurysmal rupture, although difficult to perform but, will be of high value. The incidence of development of seizures is comparatively low (2%) in patients who have been treated for SAH with surgical clipping [11], but in our case, clipping, from the diagnosis of the first aneurysm till its progression to SAH, was never performed. It was ultimately performed after the diagnosis of a fresh aneurysm on CTA scan. Patients with SAH are started on prophylactic treatment of anti-epileptics normally. However, the use of a specific class and duration of an anti-epileptic drug is still a matter of debate, and no clear guidelines are available in this regard [11,13]. Our patient was not prescribed any prophylactic drugs after SAH but when presented with a state of tonic-clonic seizures, he was put on double IV antiepileptics (sodium valproate and levetiracetam) for initial stabilization. After that, on discharge, he was put on an oral long-term course of the same anti-epileptics.

## Conclusions

In conclusion, patients with adult PKD can present with symptoms of epilepsy. It is because of the dire neurological vascular complications associated with this disease. Scar epilepsy is a rare complication of ADPKD. There are enough data available in the literature for the association between ADPKD and SAH; similarly, SAH leading to development of seizures has also been documented but cases reporting SAH leading to epilepsy, as an ultimate complication of an inherited kidney disease (ADPKD), all happening in series in a single patient, are rather limited. As the knowledge is limited, we could not ascertain with confidence which method of management for an ominous complication, like this one, is the most beneficial. We are hopeful if complex cases like this were acknowledged and brought to light, it would prove highly fruitful for the availability of better treatment strategies and outcomes for patients with ADPKD.
